# P-526. The Role of Nutritional Status and Body Mass Index in Predicting Dengue Severity in Pediatric Patients: A Logistic Regression Analysis

**DOI:** 10.1093/ofid/ofaf695.741

**Published:** 2026-01-11

**Authors:** Mohammad Abdul Hadi, Mehnaaz Sameera Arifuddin, Mohammad Abdul Hannan Hazari

**Affiliations:** Deccan College of Medical Sciences, Hyderabad, Telangana, India; Deccan College of Medical Sciences, Hyderabad, Telangana, India; Deccan College of Medical Sciences, Hyderabad, Telangana, India

## Abstract

**Background:**

Obesity is a known risk factor for infections like dengue, but its link to severity in children is less explored. This study evaluated 100 dengue infected children ( ages 2-15 ) for a correlation between body weight/BMI and disease severity. Logistic regression showed significant results (Group 1 : p = 0.002; Group 2 : p=0.000), correctly classifying over 85% of cases. Higher BMI was linked to milder disease, and male children in Group 1 had lower severity (p = 0.038). Findigs suggest good nutrition may reduce severe dengue risk.

**Methods:**

This observational study, conducted at Deccan College of Medical Sciences, Hyderabad, enrolled children aged 2-15 diagnosed with dengue (IgM-positive). Exclusion criteria included co-infections and chronic liver disease. Anthropometric data (height, weight) were recorded and BMI was calculated. Dengue severity was assessed based on WHO criteria. Logistic regression assessed the relationship between BMI/Weight and severity using SPSS 17.0 (p < 0.05).

**Results:**

100 children were included; no significant gender-based differences in anthropometric and blood parameters. Logistic regression divided children into two age groups: Group 1 (2-5 years) and Group 2 (5-15 years). Higher BMI and male gender in Group 1 were linked to lower severity. In Group 2, BMI was a significant predictor of severity (OR= 0.564, p < 0.001). Better nutritional status was associated with milder dengue.

**Conclusion:**

This study highlights the protective role of good nutritional status in dengue outcomes, with BMI serving as a potential clinical marker for risk assessment. Early identification of at-risk individuals could improve management strategies.

**Disclosures:**

All Authors: No reported disclosures

